# Krukenberg’s Spindle-Like Corneal Changes in a Patient With Prolonged ICU Admission: A Case Report

**DOI:** 10.7759/cureus.85260

**Published:** 2025-06-02

**Authors:** Yuto Shiotani, Daiyu Kosen, Keiki Shimizu, Akiko Ohno-Tanaka

**Affiliations:** 1 Department of Ophthalmology, Tokyo Metropolitan Tama Medical Center, Tokyo, JPN; 2 Department of Emergency and Critical Care Medicine, Tokyo Metropolitan Tama Medical Center, Tokyo, JPN

**Keywords:** corneal change, intensive care unit, krukenberg's spindle, pigment dispersion, post-icu ophthalmological care

## Abstract

Krukenberg's spindles are vertical, spindle-shaped pigment deposits on the posterior cornea and are primarily associated with pigment dispersion syndrome (PDS). We present a case of Krukenberg's spindle-like corneal changes in a 39-year-old male patient following prolonged ICU management for thyroid storm complicated by infective endocarditis. The patient's complex ICU course included mechanical ventilation, veno-arterial extracorporeal membrane oxygenation (VA-ECMO), and multiple medications. Ophthalmological examination revealed bilateral, brownish, linear changes in the central corneal stroma and a normal endothelial cell count. Various conditions, including drug-induced keratopathy and systemic diseases, were excluded. The findings presented a distinctive pattern of Krukenberg's spindle-like corneal changes following a prolonged ICU stay. While morphologically resembling classic Krukenberg's spindle, the condition had clinical features differing from those of typical PDS. Although the exact mechanism remains unclear, multiple factors in the ICU environment, such as the effects of medication on pupillary dynamics, thyroid storm-associated autonomic changes, patient positioning, and systemic conditions, may have contributed to causing this variant of pigment dispersion. This report emphasizes the need for ophthalmological evaluation following an extended ICU stay.

## Introduction

Corneal change can result from various causes. For example, Krukenberg’s spindles, seen in pigment dispersion syndrome (PDS), form a characteristic pattern of pigment deposition encountered in corneal changes [1-4]. While progress in critical care medicine has improved the survival rate of severely ill patients [5], there are scarce reports of corneal changes associated with a prolonged ICU stay. This report describes a unique case of bilateral, Krukenberg’s spindle-like corneal changes that developed after extended ICU management for thyroid storm complicated by infective endocarditis. Only a few corneal anomalies associated with the ICU environment have previously been described, and ophthalmological monitoring is vital in critically ill patients. To the best of our knowledge, this report is the first to describe Krukenberg’s spindle-like corneal changes associated with prolonged ICU management.

## Case presentation

The patient was a 39-year-old male with a past medical history of Graves’ disease and atopic dermatitis. His ophthalmological history was unremarkable; he wore soft contact lenses, for which he had been undergoing regular eye examinations. He had no previous history of eye disease. He had been receiving routine contact lens examinations at another ophthalmology clinic, but had not visited it recently before his current hospitalization. His previous visits had revealed no ophthalmological abnormalities, although the details could not be ascertained as he had not recently undergone a detailed examination. Before his current illness, his activities of daily living had been normal, and his health had been generally good. On July 31, 2023, the patient presented to the emergency department with a fever and altered mental status. Initial tests revealed that he was experiencing a thyroid storm complicated by infective endocarditis with marked mitral valve vegetation secondary to *Staphylococcus aureus *bacteremia. Due to the severity of his condition, he was admitted to the ICU for further management. Following stabilization in the ICU and social rehabilitation, he presented to our ophthalmology department in April 2024 with a mild, subjective decrease in vision.

The patient’s ICU management was complex and required advanced circulatory management, including the use of veno-arterial extracorporeal membrane oxygenation (VA-ECMO) and an intra-aortic balloon pump (IABP) from July 31 to August 9 for the treatment of severe shock. He received several medications, including broad-spectrum antibiotics, such as vancomycin, cefazolin, and meropenem, as well as specific treatments for the thyroid storm, such as methimazole, Lugol’s solution (5% iodine and 10% potassium iodide in distilled water), hydrocortisone, and β-blockers. Following a tracheostomy, he required ongoing respiratory management and continuous hemodiafiltration (CHDF). His hospital course was further complicated by the development of septic emboli, which resulted in multiple cerebral infarctions and symmetrical peripheral gangrene in his extremities, requiring the amputation of multiple digits. Additionally, he developed pressure ulcers and an inferior vena cava thrombus related to the ECMO cannulation. Despite these complications, his condition gradually improved, and he was eventually transferred to a rehabilitation facility. His ICU management required multiple medications for supportive care and treatment of his various conditions. Table 1 shows a detailed list of all the medications administered during his ICU stay, none of which are known to cause linear corneal stromal changes.

**Table 1:** Medications administered during ICU stay A comprehensive list of medications administered to the patient during his ICU stay. None of these medications has previously been associated with the development of corneal stromal changes like those observed in the present case ICU: intensive care unit

Start date	End date	Medication	Route of administration
July 31	September 27	Dimethyl isopropylazulene	Topical
July 31	October 10	Iodine and potassium iodide	Oral
August 2	September 27	Zinc oxide	Topical
August 3	October 1	White petrolatum	Topical
August 6	September 4	Heparinoid	Topical
August 7	September 5	Sodium hyaluronate	Topical
August 7	November 6	Sulfadiazine silver	Topical
August 11	September 9	Ofloxacin	Topical
August 12	August 17	Naldemedine tosilate	Oral
August 14	October 24	Gentamicin sulfate	Topical
August 15	October 16	Bisoprolol	Patch
August 15	October 31	Vidarabine	Topical
August 18	November 1	Acetaminophen	Oral
August 19	November 6	Lemborexant	Oral
August 20	October 22	Clostridium butyricum	Oral
August 20	November 6	Lansoprazole	Oral
August 22	October 8	Potassium gluconate	Oral
August 25	October 4	Warfarin potassium	Oral
August 25	October 10	Thiamazole	Oral
August 31	November 6	Trazodone hydrochloride	Oral
September 2	November 6	Sodium gualenate hydrate and sodium bicarbonate	Oral
September 12	September 12	Risperidone	Oral
October 11	October 18	Calcium lactate hydrate	Oral
October 11	November 6	Alfacalcidol	Oral
October 11	November 6	Levothyroxine sodium hydrate	Oral
October 12	October 12	Cefalexin	Oral
October 15	October 15	Glucose	Oral
October 15	October 24	Edoxaban tosilate hydrate	Oral
October 20	November 6	Sucrose and povidone-iodine	Topical
October 20	November 6	Betamethasone valerate and gentamicin sulfate	Topical
October 20	November 6	Hydrocortisone butyrate	Topical
July 31	July 31	Ceftriaxone sodium hydrate	Intravenous injection
July 31	July 31	Cefepime dihydrochloride hydrate	Intravenous injection
July 31	July 31	Epinephrine	Intravenous injection
July 31	July 31	Sodium bicarbonate	Intravenous injection
July 31	July 31	Electrolyte solution (IV fluid)	Intravenous injection
July 31	July 31	Ketamine hydrochloride	Intravenous injection
July 31	July 31	Fursultiamine hydrochloride	Intravenous injection
July 31	August 1	Rocuronium bromide	Intravenous injection
July 31	August 3	Vancomycin hydrochloride	Intravenous injection
July 31	August 5	Vasopressin	Intravenous injection
July 31	August 6	Calcium gluconate hydrate	Intravenous injection
July 31	August 6	Dobutamine hydrochloride	Intravenous injection
July 31	August 11	Noradrenaline	Intravenous injection
July 31	August 12	Midazolam	Intravenous injection
July 31	August 12	Glucose (IV fluid)	Intravenous injection
July 31	August 13	Ringer’s acetate (IV fluid)	Intravenous injection
July 31	August 18	Hydrocortisone sodium phosphate	Intravenous injection
July 31	August 18	Fentanyl citrate	Intravenous injection
July 31	August 18	Landiolol hydrochloride	Intravenous injection
July 31	August 19	Omeprazole sodium	Intravenous injection
July 31	October 6	Meropenem hydrate	Intravenous injection
July 31	October 10	Cefazolin sodium	Intravenous injection
August 1	August 8	Thiamine disulfide phosphate, pyridoxine hydrochloride, and cyanocobalamin	Intravenous injection
August 1	October 11	Ringer’s acetate (IV fluid)	Intravenous injection
August 2	October 10	Heparin sodium	Intravenous injection
August 7	August 23	Potassium chloride	Intravenous injection
August 9	October 27	Propofol	Intravenous injection
August 14	August 20	Dexmedetomidine hydrochloride	Intravenous injection
July 31	August 24	Thiamazole	Intravenous injection
August 18	August 20	Antithrombin gamma	Intravenous injection
August 19	August 19	Menatetrenone	Intravenous injection
August 19	August 24	Cefepime dihydrochloride hydrate	Intravenous injection
August 20	September 16	Magnesium sulfate hydrate	Intravenous injection
August 20	August 25	Acetaminophen	Intravenous injection
August 21	August 23	Potassium chloride	Intravenous injection
August 24	October 13	Cefmetazole sodium	Intravenous injection
August 25	August 25	Furosemide	Intravenous injection
August 27	August 27	Dibasic sodium phosphate hydrate sodium	Intravenous injection
August 31	September 15	Cefazolin sodium	Intravenous injection
September 8	September 8	Remifentanil hydrochloride	Intravenous injection
September 8	September 12	Electrolyte solution (IV fluid)	Intravenous injection
September 9	September 9	Flurbiprofen axetil	Intravenous injection
September 20	September 20	Acetaminophen	Intravenous injection
October 7	October 10	Dexamethasone sodium phosphate	Intravenous injection
October 11	October 11	Metoclopramide hydrochloride	Intravenous injection
October 27	November 6	Heparin calcium	Subcutaneous injection

**Figure 1:**
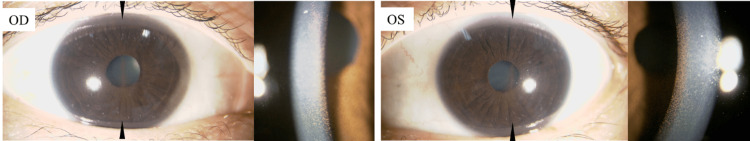
Anterior segment photographs Bilateral slit-lamp photographs demonstrating symmetrical, Krukenberg’s spindle-like corneal changes. The black arrowheads highlight the brownish, linear, stromal changes in both eyes, each measuring approximately 1 mm in width and 6 mm in length. Both eyes retained overall corneal transparency OD: right eye; OS: left eye

An ophthalmological evaluation in April 2024 found the patient’s best-corrected visual acuity (BCVA) to be 20/16 in the right eye and 20/20 in the left eye with a subjective refractive error of -6.00/1.0×160° in the right eye and -5.50/1.55° in the left eye. A slit-lamp examination revealed bilateral, linear, brownish, corneal stromal changes confined primarily to the mid-to-deep stroma of the central cornea and exhibiting a fine, punctate pattern (Figure 1).

**Figure 2:**
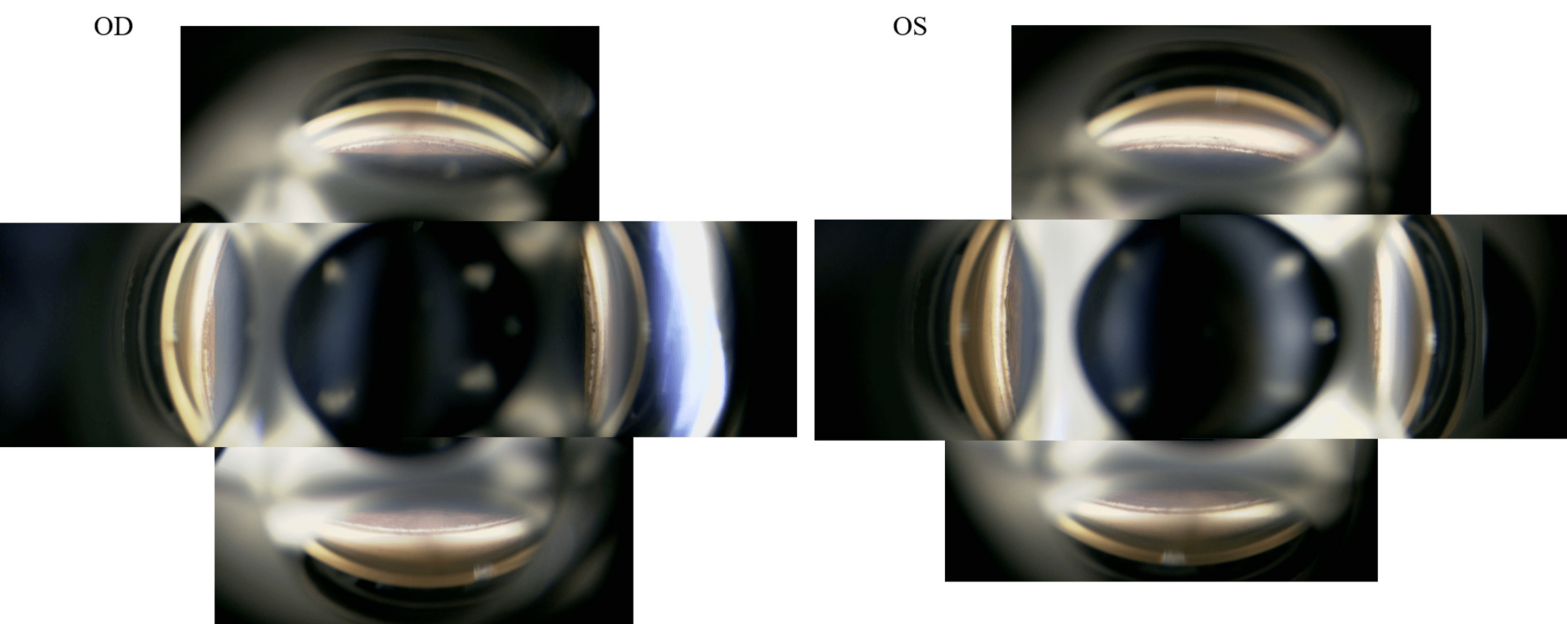
Gonioscopy images Gonioscopic examination of anterior chamber angles. Both eyes demonstrated widely open angles with mild pigmentation and consistent structural characteristics OD: right eye; OS: left eye

There was no evidence of a cataract. Gonioscopy found wide, open angles with mild pigmentation (Figure 2).

**Figure 3:**
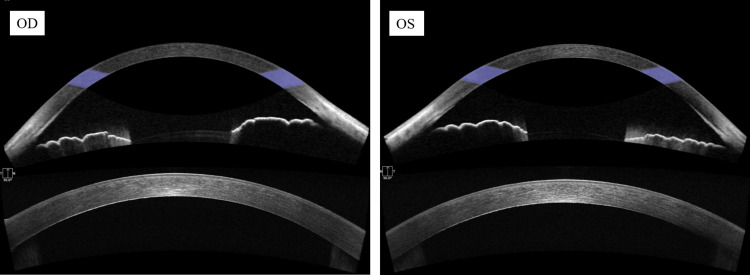
Anterior segment OCT images Bilateral anterior segment OCT images. Upper panel: iris configuration: no posterior iris bowing with open angles in either eye. Lower panel: corneal stromal analysis: hyperreflective areas corresponding to the changes observed with a slit lamp were visible in the deep stroma of both eyes, which had a normal corneal thickness and shape OD: right eye; OS: left eye; OCT: optical coherence tomography

There were no signs of anterior segment inflammation, such as keratic precipitates, cells, or a flare in the anterior chamber. Fundoscopic examination found no abnormalities of the retina, optic disc, or retinal vasculature in either eye. Dynamic perimetry confirmed right homonymous hemianopia, which was consistent with the patient's previous cerebral infarctions. Anterior segment optical coherence tomography (OCT) demonstrated hyperreflective areas corresponding to the clinically observed changes. No significant change was found in the corneal shape or thickness (Figure 3).

**Figure 4:**
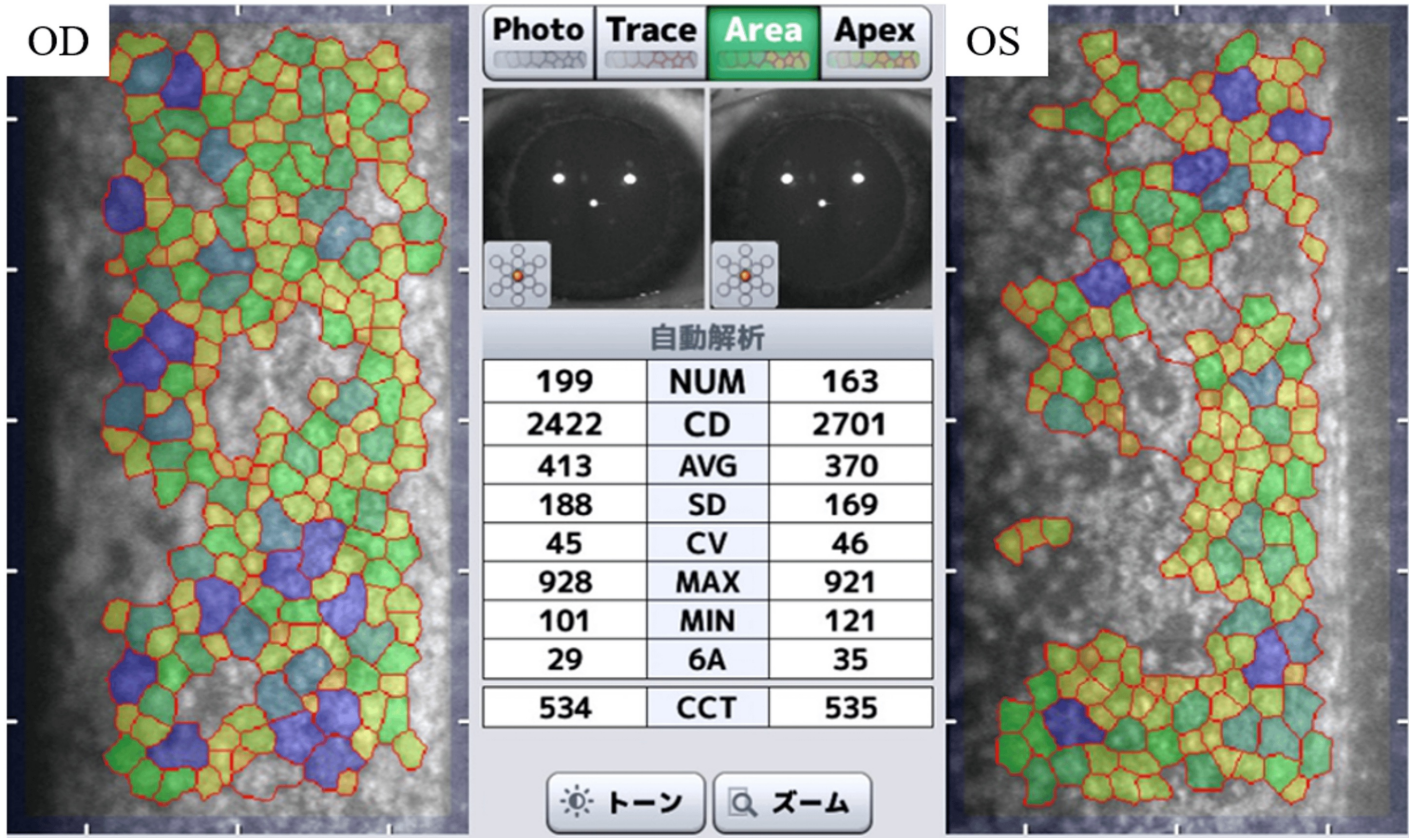
Corneal endothelial cell microscopy Specular microscopy of the corneal endothelium. Both eyes showed a normal endothelial cell count (right eye: 2,422 cells/mm²; left eye: 2,701 cells/mm²). The colors in the image, which classify individual endothelial cells based on their area, with yellow to green representing smaller areas and blue representing larger areas, are useful for visually assessing the variation in cell size. Although this automated analysis revealed variations in cell size, cellular morphology was generally normal OD: right eye; OS: left eye

Specular microscopy revealed a normal corneal endothelial cell count of 2422 cells/mm² in the right eye and 2701 cells/mm² in the left eye (Figure 4).

Laboratory tests, including a complete blood count, lactate dehydrogenase, ceruloplasmin, and serum protein electrophoresis, which were performed to exclude systemic diseases, found no abnormalities.

The patient’s extensive ICU stay and polypharmacy raised suspicion of a drug-induced corneal change. However, the causative drug could not be identified. Betamethasone phosphate ophthalmic solution was administered to decrease the stromal changes, but it produced no significant improvement. Consequently, no further treatment was administered at the time, and the patient was placed under observation. The corneal changes and visual acuity were stable at the subsequent visits.

## Discussion

This case was challenging to diagnose owing to the unique findings of bilateral, central, linear, corneal, stromal changes discovered after prolonged ICU management. Krukenberg’s spindles appear as vertical, spindle-shaped pigment deposits on the corneal endothelium and are primarily associated with PDS. In PDS, posterior bowing of the iris leads to mechanical contact with the lens-zonular apparatus, which releases pigment particles [1]. PDS presents three cardinal signs: Krukenberg’s spindles in the corneal endothelium, iris transillumination defects in the mid-peripheral region, and increased pigmentation of the trabecular meshwork [6]. Idiopathic PDS with classic Krukenberg’s spindles typically occurs bilaterally in young, myopic male patients, while secondary pigment dispersion causing Krukenberg’s spindle-like deposits has been observed following trauma, uveitis, intraocular surgery, and medication [4]. In our case, the corneal changes morphologically resembled Krukenberg’s spindles in their bilaterality and preservation of the corneal endothelial cell count, both of which align with typical PDS findings. Histologically, melanin granules reaching the corneal endothelium are incorporated into endothelial cells, and cellular function and transparency remain intact despite long-term pigment deposition [7]. However, our case was marked by the absence of iris transillumination defects and trabecular meshwork pigmentation, and the anterior segment OCT demonstrated no posterior iris bowing.

The pathophysiology of pigment dispersion is well-understood. In the reverse pupillary block mechanism, the iris acts as a flap valve, creating a pressure differential that causes posterior iris bowing and increased irido-zonular contact. Mechanical friction between the iris pigment epithelium and zonular structures during pupillary movement then results in the liberation of pigment into the anterior chamber [1-4]. We hypothesized that the unique ICU environment in our case created conditions triggering these mechanisms via novel pathways. Our patient’s thyroid storm likely played a substantial role, as this extreme, thyrotoxic state enhances sensitivity to catecholamines [8], leading to sympathetic hyperactivity affecting pupillary dynamics [9]. Recent studies have supported this association by demonstrating sympathetic predominance and bilateral mydriasis during thyroid storm [10]. Additionally, the prolonged ICU stay involved other, potential, contributory factors, including complex drug interactions, patient positioning, systemic inflammation, and changes in ocular surface homeostasis that may have further promoted abnormal pupillary dynamics and pigment dispersion.

A key, contributory mechanism was the pharmacodynamic interactions producing opposite, autonomic effects. During ICU management, the patient received sympathomimetics, such as epinephrine, which can cause mydriasis through sympathetic activation by stimulating α1-adrenergic receptors in the pupillary dilator muscle [11]. Additionally, he received sedative-analgesics, including remifentanil and midazolam, which are known to have miotic effects [12]. This drug antagonism, combined with the endogenous, sympathetic drive from the thyroid storm, likely produced anomalous iris movements, which may have increased iris-lens zonule contact, thereby promoting pigment release through mechanisms consistent with the usual pathophysiology of pigment dispersion. The combination of the thyroid storm-induced autonomic dysfunction and complex, pharmacological interactions likely created the unique circumstances that caused the distinctive corneal findings in this case.

Drug-induced keratopathy was among the foremost items in the differential diagnosis of this case. Drug-induced corneal deposits are typically bilateral and symmetrical and often improve after drug discontinuation. Brown deposits are associated with the use of chlorpromazine and rifabutin, among other medications [13]. A comprehensive review of all the medications administered during the patient's ICU stay (Table 1) revealed no previously reported association with corneal changes. Additionally, a review of past studies found no association between any of these medications and PDS or pigmentary glaucoma. While some agents may affect pupillary dynamics, no direct association with Krukenberg’s spindle formation has previously been reported.

Systemic diseases known to cause corneal changes were also considered. Various systemic conditions, such as Wilson’s disease, cystinosis, and multiple myeloma, are known to cause corneal changes [14], but the findings of the present case were not congruent with these symptoms. Normal serum ceruloplasmin and copper levels ruled out Wilson’s disease. Cystinosis was excluded as there were no systemic symptoms or urinalysis and blood test results suggestive of this disease. Multiple myeloma was deemed unlikely owing to the absence of M-protein on serum protein electrophoresis as well as the normal serum free light chain ratio. Based on all these findings, the present case was considered a unique condition related to the ICU environment: a rare form of pigment dispersion phenomenon leading to Krukenberg’s spindle-like corneal changes. While not presenting the complete clinical picture of PDS, it shared features with the latter, such as Krukenberg’s spindle-like pigment deposits and a normal corneal endothelial cell count.

This case report has several limitations. Due to the absence of detailed pre-ICU ophthalmological records, we could not confirm that the corneal changes developed de novo following a prolonged ICU stay. Therefore, the possibility that these findings were due to coincidental, pre-existing changes could not be fully ruled out. However, several factors supported an ICU-related etiology: the temporal relationship with ICU admission, the absence of classic PDS features (iris transillumination defects and trabecular pigmentation), and the unprecedented combination of multiple physiological stressors during the ICU stay that provided a plausible mechanism for activating pigment dispersion pathways.

The findings of the present case emphasize the importance of conducting an ophthalmological evaluation for patients with a history of an extended ICU stay during recovery from a critical illness. While detailed ophthalmological examinations are often difficult to perform during an ICU stay, appropriate ophthalmological monitoring during the recovery period can facilitate the early detection of corneal changes and the assessment of their impact on long-term visual function. The intraocular pressure and other relevant parameters of our present patient will be monitored using a management protocol similar to that for PDS to prevent complications, such as pigmentary glaucoma. This report offers new perspectives on the relationship between severe, systemic diseases requiring ICU management and Krukenberg’s spindle-like corneal changes.

## Conclusions

This report of bilateral Krukenberg’s spindle-like corneal changes following prolonged ICU management for thyroid storm complicated by infective endocarditis revealed features distinct from those of classic Krukenberg’s spindles and other known corneal pathologies. These findings suggest that complex, ICU-related factors likely caused the abnormal pigment dispersion. The distinctive ocular findings highlight the importance of conducting ophthalmological assessment after recovery in patients with an extended ICU stay.
